# FIB-4, APRI, and AST/ALT ratio compared to FibroScan for the assessment of hepatic fibrosis in patients with non-alcoholic fatty liver disease in Bandar Abbas, Iran

**DOI:** 10.1186/s12876-021-02038-3

**Published:** 2021-12-03

**Authors:** Behnaz Amernia, Seyed Hamid Moosavy, Fatemeh Banookh, Ghazal Zoghi

**Affiliations:** 1 Department of Gastroenterology, Faculty of Medicine, Hormozgan University of Medical Sciences, Bandar Abbas, Iran; 2 Student Research Committee, Faculty of Medicine, Hormozgan University of Medical Sciences, Bandar Abbas, Iran; 3 Endocrinology and Metabolism Research Center, Hormozgan University of Medical Sciences, Bandar Abbas, Iran

**Keywords:** NAFLD, FibroScan, Fibrosis, FIB-4, APRI, Non-invasive, AST/ALT

## Abstract

**Background:**

Non-alcoholic fatty liver disease (NAFLD) is the most common chronic liver disease worldwide. Researchers have tried to develop indices to assess liver fibrosis in NAFLD patients to avoid liver biopsy. In this study we aimed to compare fibrosis-4 (FIB-4), aspartate aminotransferase (AST) to platelet ratio index (APRI), and aspartate aminotransferase/alanine aminotransferase (AST/ALT) ratio with FibroScan for the assessment of hepatic fibrosis in patients with NAFLD.

**Methods:**

This cross-sectional study included patients with NAFLD or non-alcoholic steatohepatitis (NASH) referred to the Gastroenterology Clinic of Shahid Mohammadi Hospital, Bandar Abbas, Iran, in 2019. Demographic features of the participants including age and gender were recorded. All participants underwent FibroScan and had their AST, ALT, and platelet count measured in a random blood sample, taken within 1 month of the FibroScan.

**Results:**

Of the 205 NAFLD patients included in this study with a mean age of 42.95 ± 10.97 years, 144 (70.2%) were male. Fibroscan results revealed that 94 patients (45.9%) had F1, 67 (32.7%) F2, 29 (14.1%) F3, and 15 (7.3%) F4 liver fibrosis. A significant correlation was found between FibroScan score and FIB-4 (r = 0.572), APRI (r = 0.667), and AST/ALT (r = 0.251) (*P* < 0.001). Sensitivity, specificity, positive predictive value, negative predictive value, and accuracy of APRI at the 0.702 cut-off for the differentiation of F3 and F4 from F2 and F1 were 84.1, 88.2, 66.1, 95.3, and 87.3%, FIB-4 at the 1.19 cut-off 97.7, 72.7, 49.4, 99.2 and 78%, and AST/ALT at the 0.94 cut-off 61.4, 77, 42.2, 87.9, and 73.7% respectively. Moreover, the area under the receiver operating curve of APRI, FIB-4, and AST/ALT for the differentiation of F3 and F4 from F2 and F1 was 0.923, 0.913, and 0.720, respectively.

**Conclusions:**

Based on these results, APRI appears to be the most appropriate substitute of FibroScan for the detection of significant fibrosis in NAFLD patients. FIB-4 was the second best, suggesting that in case of FibroScan unavailability, APRI and FIB-4 are the best indices to assess liver fibrosis in NAFLD patients.

## Introduction

With an estimated prevalence of approximately 25%, non-alcoholic fatty liver disease (NAFLD) is considered an increasing public health problem, owing to its close association with type 2 diabetes mellitus, obesity, and metabolic syndrome, as well as their undeniable epidemics worldwide [1–3]. The prevalence of NAFLD among Iranians has been estimated at 33.9% in a recent systematic review and meta-analysis of 23 studies [4]. In fact, NAFLD is a spectrum of liver diseases, from fatty infiltration to steatohepatitis, fibrosis, and cirrhosis [5]. Progression of NAFLD leads to hepatic inflammation and fibrosis. Liver fibrosis is associated with an increased risk of complications, such as cirrhosis, hepatic failure, hepatocellular carcinoma, and even death [1–3]. NAFLD is also closely related to multiple significant extrahepatic manifestations, including chronic kidney disease, cardiovascular disease (CVD), and some extrahepatic cancers resulting in an increased disease burden [6]. Also, liver complications of NAFLD are expected to become the most common reason for liver transplantation in the near future. Moreover, CVD is regarded as the most common cause of mortality in NAFLD patients [1, 6].

Abdominal ultrasonography (US) is the most common imaging method for the assessment of NAFLD, with sensitivity and specificity of around 85% and 90%, respectively [7]; however, it has some limitations, including being operator-dependent and ineffective in patients with central obesity [8]. Liver biopsy is the gold standard for quantification and assessment of liver fibrosis in NAFLD patients. Not only is liver biopsy an invasive method causing pain and discomfort for the patients, but it also has rare but non-negligible complications, such as sepsis, bleeding, and damage to the surrounding structures [9]. Therefore, liver biopsy is not preferred as the first line method of evaluation and is most often reserved for patients with inconclusive results from non-invasive methods [10]. The European Association for the Study of the Liver (EASL) and the American Association for the Study of Liver Diseases (AASLD) recommend the use of transient elastography by FibroScan for the evaluation of liver fibrosis in NAFLD patients. FibroScan is a non-invasive, easy-to-use modality that can assess hepatic fat deposition and liver stiffness with high accuracy; nevertheless, this method is also limited by obesity [11–13]. Aside from FibroScan, various alternative non-invasive methods have been developed for the assessment of liver fibrosis in NAFLD during the past decade, such as aspartate aminotransferase/alanine aminotransferase (AST/ALT) ratio, fibrosis-4 (FIB-4) score, and AST to platelet ratio index (APRI) [14–16].

Given the high prevalence of NAFLD in Iran and its potential complications, the detection of this condition is very important, especially at its early stages. Moreover, non-invasive methods are preferred over liver biopsy in this regard. However, although non-invasive, FibroScan is costly and may not be available at every center. Thus, we aimed to compare FIB-4, APRI, and AST/ALT to FibroScan for the assessment of hepatic fibrosis in patients with NAFLD.

## Methods

### Participants

This cross-sectional study included patients with NAFLD or non-alcoholic steatohepatitis (NASH) diagnosed based on US findings or liver enzymes by an expert gastroenterologist according to the recommendations of the EASL, the European Association for the Study of Diabetes (EASD), and the European Association for the Study of Obesity (EASO) [17], who were referred to the Gastroenterology Clinic of Shahid Mohammadi Hospital, Bandar Abbas, Iran, during 2019. Of these patients, those who gave written informed consent to participate in the study were enrolled. This study was given ethical approval by the Ethics Committee of Hormozgan University of Medical Sciences (IR.HUMS.REC.1398.170) and it complies with the statements of the Declaration of Helsinki. Exclusion criteria were incomplete demographic or clinical information, other chronic liver diseases, including hepatitis B, hepatitis C, or autoimmune hepatitis (AIH), alcoholic liver disease, the use of hepatotoxic medications such as chronic intake of methotrexate, advanced liver disease, congestive heart failure, hepatic congestion, any condition interfering with FibroScan evaluation such as elevated body mass index (BMI), decompensated cirrhosis based on clinical or US evidence, and the use of hormonal or herbal medications. Patients were recruited through convenience sampling. The sample size was calculated as at least 200 based on the correlation coefficient of 0.23 in Fallatah et al.’s study [18], α = 0.05, β = 0.5, and the following formula:

$$n = \frac{{\left( {Z_{{1 - \frac{\alpha }{2}}} + Z_{1 - \beta } } \right)^{2} }}{{\left( {0.5\ln \left( {\frac{1 + r}{{1 - r}}} \right)} \right)^{2} }} = \frac{7.84}{{\left( {0.5\ln \left( {\frac{1 + 0.23}{{1 - 0.23}}} \right)} \right)^{2} }} + 3 = 200$$

### Study design

A checklist was used to record the data. First demographic features of the patients including age and gender were recorded. Then, all patients underwent FibroScan using the FibroTouch 502 device (Echosens, France). All FibroScans were performed according to the manual of the manufacturer. Based on the previous studies and the recommendations of the manufacturer, FibroScan results were classified as:

- F0: 1–6 kPa
- F1: 6.1–7 kPa
- F2: 7.1–9 kPa
- F3: 9.1–10.3 kPa
- F4: ≥ 10.4 kPa

Controlled attenuation parameter (CAP) score, showing the amount of liver with fatty change, was also determined in FibroScan for each patient. The following measurements were done in random blood samples of all patients within 1 month of the FibroScan evaluation:

- Serum ALT with 45.25 U/L as upper limit of normal in men and 30.47 in women
- Serum AST with 15–37 U/L as the normal range
- Platelet count with 150,000–400,000/µL as the normal range

Liver enzymes (AST and ALT) were measured using the Flex Reagent Cartrige (Siemens Healthcare Diagnostics, Germany).

AST/ALT ratio was calculated for each patient. APRI and FIB-4 were also calculated based on the following formulas:

$${\text{APRI}} = \frac{{\frac{{{\text{AST}}\,{\text{level}}}}{{{\text{AST}}\,{\text{ULN}}\,({\text{upper}}\,{\text{limit}}\,{\text{of}}\,{\text{normal}})}}}}{{{\text{Platelet}}\,{\text{count }}\left( {10^{9} /{\text{L}}} \right)}} \times 100$$

$${\text{FIB}} - 4 = \frac{{{\text{Age}}\,({\text{years}}) \times {\text{AST}}\,{\text{(U}}/{\text{L)}}}}{{{\text{Platelet}}\,{\text{count}}\,(10^{9} /{\text{L}}) \times \sqrt {{\text{ALT}}\,({\text{U}}/{\text{L}})} }}$$

### Data analysis

The Statistical Package for the Social Sciences (SPSS) software (version 25.0, Armonk, NY: IBM Corp.) was used for data analysis. Mean, standard deviation, median, interquartile range (IQR), frequency, and percentages were used to describe the results. Distribution normality of quantitative variables were determined using the Kolmogorov–Smirnov normality test. Accordingly, Spearman’s correlation was used to determine their correlations and the Mann-Whitney test was used for comparison by gender. The receiver operating characteristic (ROC) curves were drawn to determine the diagnostic value of FIB-4, AST/ALT ratio, and APRI for the differentiation of F1 and F2 of liver fibrosis from F3 and F4 (in FibroScan). The area under the ROC (AUROC) curve was calculated for each non-invasive index. The optimal cut-off of all three indices was also determined for this purpose using the ROC curves. Sensitivity, specificity, positive predictive value (PPV), negative predictive value (NPV), and diagnostic accuracy (DA) were calculated for these cut-offs as well. *P* values ≤ 0.05 were regarded as statistically significant.

## Results

Of the 205 patients with NAFLD or NASH included in this study, with a mean age of 42.95 ± 10.97 years, 144 (70.2%) were male and 61 (29.8%) were female. General characteristics of the study participants are shown in Table 1. Based on FibroScan results, 94 patients (45.9%) were classified as F1, 67 (32.7%) as F2, 29 (14.1%) as F3, and 15 (7.3%) as F4. The mean AST and ALT levels were 44.75 ± 22.58 and 51.57 ± 43.37 U/L, respectively. The mean platelet count was 236,414.63 ± 91,908.84/µL.

**Table 1:** General characteristics of the study participants

Variables	Values
Age (years) mean ± SD	42.95 ± 10.97
Gender N (%)	
Male	144 (70.2)
Female	61 (29.8)
AST (U/L) mean ± SD	44.75 ± 22.58
ALT (U/L) mean ± SD	51.57 ± 43.37
Platelet count (/µL) mean ± SD	236,414.63 ± 91,908.84
FibroScan score (kPa) mean ± SD	8.70 ± 5.43
CAP score (dB/m) mean ± SD	307.33 ± 47.62
APRI mean ± SD	0.629 ± 0.661
FIB-4 mean ± SD	1.45 ± 1.27
AST/ALT ratio mean ± SD	0.92 ± 0.25
FibroScan results N (%)	
F1	94 (45.9)
F2	67 (32.7)
F3	29 (14.1)
F4	15 (7.3)

*N* number, *SD* standard deviation, *AST* aspartate aminotransferase, *ALT* alanine aminotransferase, *CAP* controlled attenuation parameter, *APRI* AST to platelet ratio index, *FIB-4* fibrosis-4

A significant correlation was found between FibroScan score and FIB-4 (r = 0.572), APRI (r = 0.667), and AST/ALT ratio (r = 0.251) (*P* < 0.001). Nonetheless, the correlation of FibroScan with FIB-4 was moderate, with APRI was strong, and with AST/ALT ratio was weak. Among different indices, only FIB-4 was significantly correlated with age (r = 0.272, *P* = 0.001); however, the correlation was weak (Table 2). Comparison of different indices between men and women showed no significant difference (Table 3).

**Table 2:** Correlation of different indices with FibroScan, CAP scores, and age

First variable	Second variable	Correlation coefficient	*P* value*
FIB-4	CAP score	0.057	0.417
	FibroScan score	0.572	< 0.001
	Age	0.272	0.001
APRI	CAP score	0.046	0.511
	FibroScan score	0.667	< 0.001
	Age	− 0.142	0.090
AST/ALT ratio	CAP score	− 0.048	0.496
	FibroScan score	0.251	< 0.001
	Age	− 0.045	0.596
FibroScan score	Age	− 0.022	0.791

*AST* aspartate aminotransferase, *ALT* alanine aminotransferase, *CAP* controlled attenuation parameter, *APRI* AST to platelet ratio index, *FIB-4* fibrosis-4

^*^Analyzed by Spearman’s correlation

**Table 3:** Comparison of different indices by gender

Indices	Male (n = 144)	Female (n = 61)	*P* value*
FibroScan score (kPa) median (IQR)	7.30 (6.30–8.80)	7.20 (6.60–9.65)	0.144
APRI median (IQR)	0.43 (0.26–0.69)	0.45 (0.25–0.84)	0.577
FIB-4 median (IQR)	0.98 (0.74–1.56)	1.24 (0.84–1.95)	0.055
AST/ALT ratio median (IQR)	0.89 (0.81–0.97)	0.92 (0.84–0.97)	0.535

*IQR* interquartile range, *AST* aspartate aminotransferase, *ALT* alanine aminotransferase, *CAP* controlled attenuation parameter, *APRI* AST to platelet ratio index, *FIB-4* fibrosis-4

*Analyzed by Mann–Whitney test

**Fig. 1:**
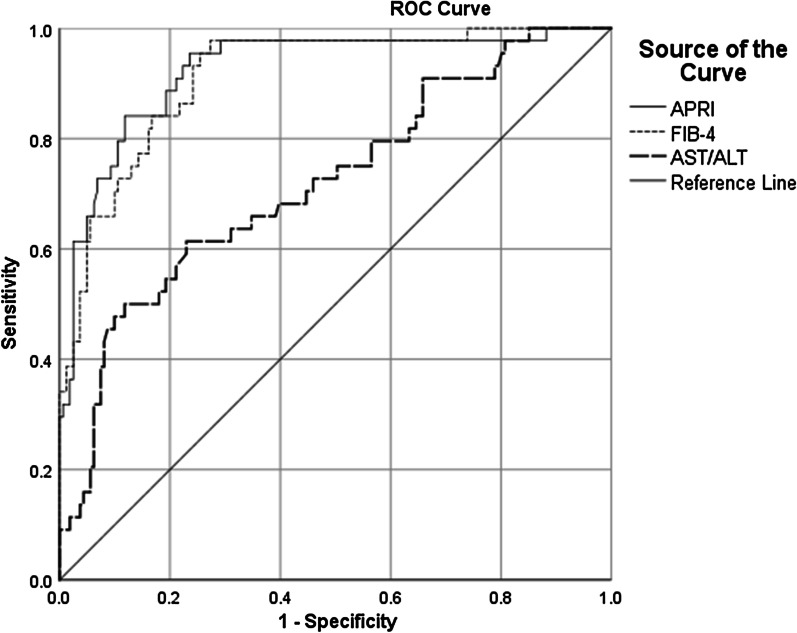
ROC curves of APRI, FIB-4, and AST/ALT ratio for the detection of F3 and F4 of liver fibrosis from the lower stages

Figure 1 demonstrates the ROC curves of APRI, FIB-4, and AST/ALT ratio for the detection of F3 and F4 of liver fibrosis from the lower stages (F1 and F2). Based on these curves, the best index to diagnose F3 and F4 from lower stages of liver fibrosis was APRI, with an AUROC curve of 0.923 (95% confidence interval [CI] 0.876–0.970). The optimal cut-off of APRI was 0.702 for this purpose, with a sensitivity of 84.1%, specificity of 88.2%, PPV of 66.1%, NPV of 95.3%, and DA of 87.3%. Results for other indices are shown in Table 4.

**Table 4:** Diagnostic performance of the indices for the differentiation of F3 and F4 from lower stages

Indices	AUC (95% CI)	*P* value	Optimal cut-off	Sensitivity (%)	Specificity (%)	PPV (%)	NPV (%)	DA (%)
APRI	0.923 (0.876–0.970)	< 0.001	0.702	84.1	88.2	66.1	95.3	87.3
FIB-4	0.913 (0.868–0.958)	< 0.001	1.19	97.7	72.7	49.4	99.2	78.0
AST/ALT	0.720 (0.631–0.808)	< 0.001	0.94	61.4	77.0	42.2	87.9	73.7

*AST* aspartate aminotransferase, *ALT* alanine aminotransferase, *CAP* controlled attenuation parameter, *APRI* AST to platelet ratio index, *FIB-4* fibrosis-4, *AUC* area under the curve, *CI* confidence interval, *PPV* positive predictive value, *NPV* negative predictive value, *DA* diagnostic accuracy

## Discussion

The results of the current study revealed APRI as the best index to differentiate F3 and F4 of liver fibrosis from F1 and F2 compared to FIB-4 and AST/ALT ratio. APRI, with an AUROC curve of 0.923 at a cut-off of 0.702, had 84.1% sensitivity, 88.2% specificity, 66.1% PPV, 95.3% NPV, and 87.3% DA for this purpose. Meanwhile, for FIB-4, the AUROC curve was 0.913 and the corresponding diagnostic values at a cut-off of 1.19 were 97.7, 72.7, 49.4, 99.2, and 78.0%, respectively. As for AST/ALT ratio, the AUROC curve was 0.720 with an optimal cut-off of 0.94, having 61.4% sensitivity, 77.0% specificity, 42.2% PPV, 87.9% NPV, and 73.7% DA.

APRI was primarily introduced by Wai et al. who showed an AUROC curve of 0.8 for advanced fibrosis (F3-F4) [19]; however, they evaluated patients with chronic hepatitis C, who were excluded from our study. AST/ALT ratio has also been primarily used in cohorts of patients with chronic hepatitis C [20]. The diagnostic accuracy of APRI and AST/ALT ratio has been reported to be low for diagnosing advanced fibrosis in patients with NAFLD (AUROC of 0.74 for the differentiation of F3 liver fibrosis) in one study [21]. The best performance of APRI for the diagnosis of significant fibrosis has been reported in patients with chronic hepatitis C, with values ≥ 1.5 predicting advanced fibrosis with a PPV of 88%. Nevertheless, weaker performance of this index has been shown in chronic liver diseases of different etiologies, including chronic hepatitis B, alcoholic liver disease, and NAFLD with AUROC of 0.72, 0.59, and 0.73, respectively [22]. The higher diagnostic performance of APRI in our study, contrary to previous findings, can be due to the measurement accuracy of laboratory parameters in the APRI formula, as well as NAFLD as the etiology of fibrosis in our study, and taking FibroScan results instead of biopsy findings as the reference of fibrosis staging. However, FibroScan has been recommended by the EASL and the AASLD for the assessment of liver fibrosis in NAFLD patients due to its non-invasiveness, ease of use, and high accuracy. The only limitation of FibroScan appears to be obesity [11–13]. Yet, a recent study reported that FIB-4 and APRI are valuable for excluding advanced fibrosis in morbidly obese patients with NAFLD [23].

Of note, although liver biopsy has traditionally been the gold standard and reference method for evaluating liver fibrosis, it has some limitations that has made its use questionable. This can also be the reason for the difference between the findings of previous studies and our results regarding the diagnostic performance of non-invasive indices, as they have most commonly taken the results of biopsy as reference, while we used FibroScan results. One limitation of liver biopsy is that a small volume of liver is evaluated which cannot reflect the fibrotic changes in the entire liver. Another limitation is that various parts of the liver may be at different stages of liver fibrosis and the extracted sample may not be indicative of the true stage of fibrosis. Moreover, biopsies are evaluated by pathologists which makes their experience an influential factor in the assessment of fibrosis [15].

FIB-4 and APRI have been recommended by many guidelines, including the World Health Organization (WHO) guidelines to determine the stage of fibrosis in countries with limited resources [24–26]. A retrospective study of 113 chronic hepatitis C patients has demonstrated good diagnostic performance of APRI and FIB-4 for determining advanced fibrosis and cirrhosis in hepatitis C patients [27]. Moreover, in a systematic review by Lee et al., FIB-4 and APRI were comparable with liver biopsy in terms of risk stratification for liver-related morbidity and mortality. Nonetheless, NAFLD fibrosis score (NFS) had the same properties in their study [28]. The high NPV of these two indices in our study (99.2% and 95.3%, respectively), suggests that they can be used to exclude advanced fibrosis in NAFLD patients. Nonetheless, AST/ALT ratio had a lower NPV. McPherson et al. have also reported a high NPV for AST/ALT ratio and FIB-4 (93% and 95%, respectively) [16].

FIB-4 was the only index significantly correlated with age in our study. This is because only FIB-4 includes age in its formula. This index was first used by Sterling et al. for the evaluation of liver fibrosis in patients with hepatitis C coinfected by human immunodeficiency virus. These researchers showed an AUROC of 0.765 for FIB-4 to identify advanced fibrosis [29]. FIB-4 has also been validated for the detection of significant fibrosis in isolated hepatitis C and B infections with AUROCs of 0.85 and 0.81, respectively [30, 31]. However, contradictory to our findings, FIB-4 has been reported to have better performance compared to APRI in NAFLD [16, 32].

In the current study, AST/ALT ratio had the lowest diagnostic performance compared to FIB-4 and APRI to differentiate mild to moderate from advanced fibrosis. This is in line with the findings of Fallatah et al. [18]. They also compared APRI, FIB4, and AST/ALT ratio for the diagnosis of significant fibrosis in NAFLD patients, suggesting APRI and FIB-4 scores to be used in the follow-up of NAFLD patients at early stages with no clear indication for liver biopsy.

## Conclusions

We found APRI to be the best index to predict advanced liver fibrosis compared to FIB-4 and AST/ALT ratio, with this index having the strongest correlation with FibroScan results. Therefore, in the setting of limited resources where FibroScan is not available, APRI is an appropriate index for the prediction of significant liver fibrosis, contributing to decision making for further evaluations, referral to higher levels, and potentially lifestyle modifications or prescription of medications.

## Data Availability

The datasets used and/or analyzed during the current study are available from the corresponding author on reasonable request.

## Abbreviations

AASLD: American Association for the Study of Liver Diseases
AIH: Autoimmune hepatitis
ALT: Alanine aminotransferase
APRI: AST to platelet ratio index
AST: Aspartate aminotransferase
BMI: Body mass index
CAP: Controlled attenuation parameter
CI: Confidence interval
DA: Diagnostic accuracy
EASD: European Association for the Study of Diabetes
EASL: European Association for the Study of the Liver
EASO: European Association for the Study of Obesity
FIB-4: Fibrosis-4
NAFLD: Non-alcoholic fatty liver disease
NASH: Non-alcoholic steatohepatitis
NPV: Negative predictive value
PPV: Positive predictive value
SPSS: Statistical Package for the Social Sciences
